# Transfemoral transcatheter aortic valve implantation for bicuspid aortic stenosis with tortuous aorta after total arch replacement

**DOI:** 10.1007/s12928-020-00738-3

**Published:** 2020-12-07

**Authors:** Daisuke Hachinohe, Hidemasa Shitan, Umihiko Kaneko, Ken Kobayashi, Keijiro Mitsube, Ryuji Koushima

**Affiliations:** 1 Cardiovascular Medicine, Sapporo Heart Center, Sapporo Cardio Vascular Clinic, 8-1, Kita-49 Higashi-16, Higashiku, Sapporo, 007-0849 Japan; 2 Cardiovascular Surgery, Sapporo Heart Center, Sapporo Cardio Vascular Clinic, Sapporo, Japan

**Keywords:** Transcatheter aortic valve implantation, Bicuspid aortic stenosis, Tortuous aorta, Total arch replacement

A 72-year-old woman with a history of hypertension, diabetes mellitus, dyslipidemia, atrial fibrillation, and cardiogenic cerebral embolism had undergone total arch replacement 9 years ago. Recently, she has experienced exertional dyspnea. Transthoracic echocardiography revealed very severe aortic stenosis (AS) with an aortic valve (AV) area of 0.74 cm^2^, mean transaortic pressure gradient of 70 mmHg, and preserved left ventricular (LV) ejection fraction of 61%. Transesophageal echocardiography (TEE) and multidetector computed tomography imaging revealed type 0 calcified bicuspid AV based on the Sievers classification (annulus area: 478mm^2^ and inter-commissural length: 38.5 × 29.3 mm) (Fig. 1, panel A). The thoracoabdominal aorta showed tortuosity (Fig. 1, panel B), and her sinus of Valsalva of 35.0 × 41.0 mm in diameter was large compared with an artificial aortic graft of 20.6 × 24.7 mm, and the aortic angle was 55° (Fig. 1, panel C). Because of high frailty (clinical frailty scale score: 6 and short physical performance battery score: 5 points), the patient was referred for transfemoral transcatheter aortic valve implantation (TAVI).

**Fig. 1:**
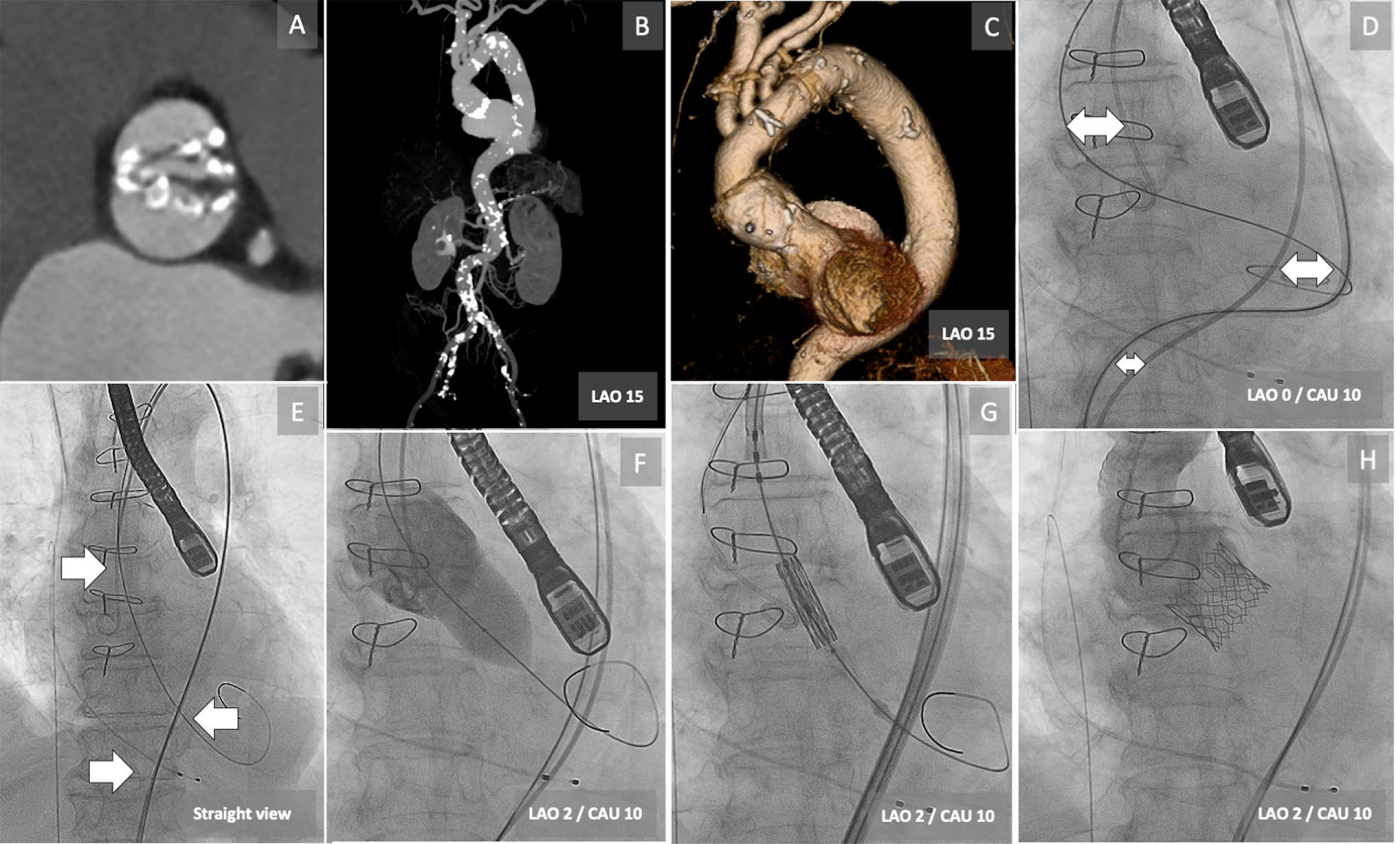
Transfemoral transcatheter aortic valve implantation (TAVI) for bicuspid aortic stenosis with a tortuous aorta after total arch replacement. **a** 3D computer tomography (CT) reconstruction shows type 0 calcified bicuspid aortic valve. **b** The tortuous thoracoabdominal aorta. **c** The synthetic prosthesis of the total arch replacement and the large ascending aorta. **d** Catheter prolapses (double-headed allows) at the ascending part to the arch graft and the tortuous thoracoabdominal aorta (Online video 1). **e** Straighten the catheter using double stiff guidewires (allows). **f** Balloon aortic valvuloplasty with 23-mm balloon. **g** Easy crossing of SAPIEN 3 through the aorta and aortic valve [Online video 2]. **h** Good result after TAVI without any complications

The TAVI was performed under general anesthesia. After placing a Safari^2^ (Boston Scientific, USA) small curve wire in the mid LV, balloon aortic valvuloplasty (BAV) was tried using Z-MEDII 23 × 40 mm (B BRAUN, USA) to make it easier to cross the device due to very severe AS and to confirm the valve size in type 0 bicuspid AV. However, the balloon could not cross to AV because of strong resistance at the ascending part to the arch graft and the tortuous thoracoabdominal aorta (Fig. 1, panel D, online video 1). Consequently, we changed the Safari^2^ wire to a Lunderquist Extra Stiff (Cook Medical, USA) and advanced another stiff guidewire through a 5 French pigtail catheter (Fig. 1, panel E). Then, the 23-mm balloon was crossed without resistance and BAV was performed (Fig. 1, panel F). A 26-mm SAPIEN 3 (Edwards Lifesciences, USA) was easily crossed through the aorta and AV (Fig. 1, panel G, online video 2), and implanted during rapid ventricular pacing with underfilling volume of a delivery balloon. TEE and aortography revealed neither significant aortic regurgitation nor other potential complications (Fig. 1, panel H).

This is the first case report showing the feasibility of transfemoral TAVI in patients with bicuspid AS after total arch replacement with a tortuous aorta. Generally, in patients with extremely tortuous access root, the buddy wire technique is useful [1]. In this case, the patient had a bicuspid AS, which is usually associated with aortopathy and leads to an ascending aortic aneurysm. The diameter difference between the enlarged aortic root and narrow artificial aortic graft, and the extremely tortuous aorta could have represented a resistance point that led to difficulty in manipulation of the catheter. The buddy stiff guidewire could straighten the access root and reduce resistance points, leading to a greater pushing force against the resistance force. Transfemoral TAVI with buddy wire technique could be feasible for treating bicuspid AS after total arch replacement with a tortuous aorta.

## Supplementary Information

Below is the link to the electronic supplementary material.

Online video 1: Catheter prolapses (double-headed allows) at the ascending part to the arch graft and the tortuous thoracoabdominal aorta.1 (MP4 7148 KB)

Online video 2: Easy crossing of SAPIEN 3 through the aorta and aortic valve.2 (MP4 9670 KB)
